# The Surgical Solution of the Problem of Race Culture*Presented to the General Session and Memorial Exercises, State Medical Association of Texas, Amarillo, May 9, 1911.

**Published:** 1913-06

**Authors:** Malone Duggan

**Affiliations:** San Antonio, Texas


THE
### TEXAS MEDICAL JOURNAL.
Established July, 1885

F. E. DANIEL, M. D., - - - - - Editor, Publisher and Proprietor

Published Monthly.—Subscription, $1.00 a Year.

VOL. XXIX. AUSTIN, JUNE, 1913. No. 12.

The publisher is not responsible for the views of the contributors.

For Texas Medical Journal.

### Original Articles.

### The Surgical Solution of the Problem of Race Culture.*

BY MALONE DUGGAN, M. D., SAN ANTONIO, TEXAS.

THE FUNCTIONS OF THE GOVERNMENT.

Obviously, the first duty in considering any question is to inquire into the principles on which it is predicated. So, regarding this question we will analyze the premises on which our conclusions are based.

First, then, we will consider the function of government—the role government plays in man's social evolution. Government, in principle, is by man and for man. It is necessary for his protection and best development; its scope is as extended as human thought and action. It is designed to secure for him the greatest degree of happiness and prosperity. It reaches its highest function when it permits, unimpeded, the highest mental, moral and physical development. The laws and conventions established by government at any given period are what the scientific achievements of that particular time make them. Man, in other words, determines his own folk lore. Therefore, it is the function of government to encourage social progress and to protect her citizenship from retrograding influences. It is out of this primal principle that government derives her right to compel normal development.

As an example, it is seen that ignorance causes failure and much unhappiness, therefore government fosters education. Disease and pestilence decimates and weakens the race; therefore, government

*Presented to the General Session and Memorial Exercises, State Medical Association of Texas, Amarillo, May 9, 1911.

establishes sanitary regulations. Disobedience to law is demoralizing and might inflict personal injury or property loss; therefore, government maintains police regulations. Control of natural resources and public utilities is necessary to prosperity; therefore, government provides regulations for these. In fact, its function is as broad as human needs, and will progress as long as it does not impede normal healthy growth.

Government is essentially co-operative. There is no such thing as a separate human being; each individual must contribute his part, his work, his thought, and that government is the best where the individual makes the largest personal sacrifice for the good of the whole. Personal liberty does not permit a man to do and act as he pleases when such actions affect his own welfare or the personal rights of others.

Comparison of modern governments with these rules of social ethics will reveal a terrible deficiency from the standard. For, although tremendous strides have been made in our social progress, the arts, trades and commerce, the moral conscience has decayed. The innate man, that which endures, has not advanced for over two thousand years. This is demonstrated by the great amount of unhappiness, suffering and depravity seen in every walk of life.

### MORAL CHARACTER AND SEX.

Strong character depends upon the refinement of a man's sexuality. Morality, generally, is associated in the minds of people with some idea of sex. For this reason it is often confounded with the vulgar or some immoral practice. This error leads to much confusion in discussing any moral question. This association comes out of the fact that the sex function predominates over all other instincts and is essentially of the greatest interest to man. People of the past have persistently misunderstood this vital force. The subject has been too personal for free discussion, and absolute license has been the custom. The fallacious idea that the sex function was only intended for the purpose of reproduction, and that beyond this its exercise was immoral, has done much to subvert the intention of this function and to break down our ethical codes.

To science we are due a new conception of sex, and we are now emerging into a new sex dispensation. We have found the key to the fuller development of the moral man. So armed, we will proceed to reconstruct our ethics on new lines as to better control the inherent nature of man.

This new dispensation teaches that "sex is the largest and most complex, the most important and interesting of all human themes."

It elevates the importance of man as distinguished from brute creation. "He brings forth a being endowed with immortality, a creature who is to be a conscious personality, to think, to suffer and enjoy, to will; a social entity, which shall effect for good or evil his fellow creatures." "Around it centers the highest aspirations, the noblest sentiment, the loftiest ideals, the purest devotion of which humanity is capable." On its virility and considerate exercise depends the happiness of the home and the stability of the nation. It was given to man to sensatize growth into vigorous manhood and womanhood; to develop the masculine character of man and the subtle charms of woman; to command respect and adoration and make love sublime. Stifle this instinct, abuse its functions, or permit it to be uncontrolled and death is the penalty, or far worse, depravity and remorse.

This false conception has forbidden parents and teachers from instructing the child and youth in the nature and purpose of his iridescent feelings. They have labored under the impression that anything pertaining to sex was impure and vulgar. This explains why our knowledge of sexuality has been so perverted and accounts for the presence of venereal diseases and the neurotic conditions that have now honeycombed the race. Darkness nourishes the mysterious; groping blindly, never results in order. So out of the disorder of our sexual knowledge the noble purpose of man has been all but destroyed.

The venereal peril is responsible for more suffering and incapacity than all other diseases combined. Its morbidity and mortality are daily increasing. It destroys the happiness of the home by levying tribute on the innocent wife and child; it lessens the earning capacity of the father who must provide for a dependent family; it saps the vitality of the youth from whom the future generations must spring; it vitiates the moral conscience on which character and stability depend; it is the undoing of nature's design, the development of a vital race.

### HEREDITY AND THE BASIC PRINCIPLE.

As individuals we are a link in a chain connecting the vitalized and organized history of our ancestors with the unborn generations of the future. Our capacities and infirmities are just what others have capitalized and bequeathed to us. And our children will be, in a large measure, just what they can develop out of the inherent qualities we transmit to them. This is made clear by the Mendelian laws of heredity which show how definite characters are formed in the individual and how they are transmitted. By knowing what similar unit characteristics both the parents lack, what both possess and in which characters they differ; by knowing of each character whether it is due to the presence or absence of a determiner, it is possible, in a relative degree, to determine what the characteristics will be in a given organism. This theory furnishes the scientific basis for the future control of the race.

The significance of this law is appalling when we realize its far-reaching import. It means that the present generation is responsible for the mental, moral and physical standard of the next, and presents in a startling manner the personal responsibility of parenthood. While the supreme purpose of all life forms is reproduction, nature never intended that the laws of selection and survival of the fittest should be artificially controlled by man. With him, in the exercise of his free moral agency, this has a far more transcendental meaning. For he not only must answer before the bar of social justice, but the honor and love of his children will be measured by the heredity he bequeaths to them. This is what Heins meant when he said that a man should be very careful how he selected his parents.

The individual has not felt this responsibility, and society has failed to exercise her right of government by controlling the racial integrity of her people.

The insane, the feeble-minded, the chronic inebriate, the criminal, the prostitute and the sexual pervert, are such because of direct inheritance, or weakened nervous systems transmitted from degenerate strains. Latent defects in the germ plasm results in degeneracy. Again, degenerate strains lessen the resistance of the germ plasm to the influence of the racial poisons and therefore increases the susceptibility of the organism to disease. This explains why tuberculosis and syphilis have so greatly depleted our physical strength; why neurasthenia, hysteria, and psychoasthenia are sapping the nervous vitality of the race.

Society heretofore has depended on environments and education to correct these errors. That this has failed is proven by the rapidly increasing rate of degeneracy. Environment ameliorates suffering and increases capacity. No doubt many with criminal and perverted strains have been prevented from becoming themselves criminals and perverts in fact, but they are criminals and perverts still, in that they transmit the same strains to their children, who, under less favorable environments, will develop the same morbid characters. Environment can, however, by conscious effort, diminish the deterrent element by breeding the defective strain to sound stock. So, likewise, they can continue to multiply by permitting the same breeds of lower strains to procreate. The child does not inherit its father's defects, but both alike inherit from the same strain—"a chip off of the old block."

The non-resistance of the protoplasm in the cell is due to the absence from the organism of a normal character. This defect may occur in any particular structure of the body or in the entire organism, and explains how unfavorable environments will affect those more severely, who by heredity have the least resistance.

There are now in the United States over half a million defectives cared for by institutions and charities. A larger number still is supported by relatives and friends. This taxes the government over $100,000,000 annually. The rate of increase of the criminal classes in 1850 was one to 3442. Now it is one to about every 600. In England the proportion of degeneracy is one to every 85 of her population. Over 65,000 of this class are either married or have been. In this State there were, in 1860, one insane person to every 12,800 population. Last year the proportion was one to every 450. The expense to the State for their keeping was $1,000,000, and this amount will increase every year. Dr. Danna, according to Dr. Barker, places the rate of degeneracy in this country at 4 per cent annually.

Are these not startling facts? And is not to know them enough to make every lover of humanity, every patriot of his beloved country, rise up in his might and do what he can to correct the evil?

### VASECTOMY AND FALLECTOMY.

Education is doing much to undo what prejudice and ignorance has caused. The same free moral agency that will permit man to destroy himself will also allow him to rise above his environment. So education today is exercising, unconsciously, the law of selected mating. A well intentioned man does not mean to inflict suffering and degradation on his innocent wife, much less to transmit degeneracy to his more innocent child. There still remains sufficient moral stamina in the race, if properly directed, to blot out half of our degeneracy in one generation, and revolutionize our social and economical status to a degree not dreamed of.

From what we have seen as belonging to the function of government, society clearly has the right to exercise this moral reform. First of all, education is essential to impress man with the sense of his responsibility. But this alone is futile, as it does not take into consideration the biologic principle for the cause of this deterioration. For like reasons, all laws attempting to regulate and control crime and prostitution are of no avail. The proposition is this: Deterioration of the race, criminality, prostitution, sexual perversion and non-resistant protoplasm are inherent strains in the organism. While environment can do much to make them tolerable, it does not and can not wholly eradicate them. Therefore, we are thrown back on the absolute alternative of checking their continuation by the regulation of the reproductive function. Now, if every individual could be relied upon to intelligently exercise his right to choice, this reform could be accomplished without government interference. But society owes too much to itself, and degeneracy is too extended in its effects, to depend on this. Until the chain is once broken, and selected mating is consciously done, no relief from this cause can be expected.

There is but one solution, and that is for the government to assume control and hold every man accountable for the character of his offspring. Utopian schemes for racial reforms have been from time to time proposed, and have failed because they did not take into account the real basic principle of life—the nature of the sexual instinct and the necessity for it to exercise its choice. Nor will any government control that likewise disregards this inalienable right succeed. Such as municipal regulation of prostitution, segregation of the unfit or the deliberate breeding of only the strong. Man will continue to love and mate so long as life lasts. And every "Jack can find his Jill." No restriction need be made to the perfect freedom to love. But the necessity for exercising judgment in the selection for marriage must be safeguarded.

Science has opened the way to the solution of government control. By the operations of *Vasectomy* and *Fallectomy*, reproduction can be effectually stopped without in the slightest way interfering with a man's or woman's sexual power or privilege of choice. Now, since the government can exercise the right to protect its subjects who can not protect themselves, and as it is universally conceded that certain classes should not be allowed to procreate, then the government should, by law, compel these operations on these classes. Who would question the moral obligation to enforce this rule now on the insane, the habitual criminal, the imbecile, the epileptic and the sexual pervert? By degrees, as society adapts itslf to such regulations, the operation could be extended to the tuberculous, syphilitic, neurasthenic and others.

What would be the result? The stock of the insane being cut off, there would be no asylums to fill. The criminal traits disappearing from the race, there would be no jails to remind man of his depravity. The sexual anomaly of man being eliminated there would be no prostitution to defile the morals of society. Instead, the race would increase by a normal growth of men and women endowed with a superior mental, physical and moral constitution.

Though this may sound altruistic at first, it is nevertheless real, and it only lacks the spark of public approval to start a conflagration of sentiment that will never stop until man shall stand forth the perfect pattern he was designed to be.

A WORD TO THE PROFESSION.

Who, besides the doctor, is better prepared to lead in this reform? By education and intimate association with the family, he can, more than any other person, educate the public.

This Association through its House of Delegates has endorsed this great movement by authorizing the organization of the Texas State Society of Social Hygiene. It was thought that more could be accomplished through a separate organization that could be more closely affiliated with the laity. This has proved to be correct. The society so authorized has now affiliated with it, in the work, over 80,000 lay workers. The fact is, the public has become so much interested that the doctor is in danger of having his vested rights and privileges taken from him. It was never intended by forming this separate society that the doctor should be eliminated. On the contrary, he was expected to be relied on to give his support and encouragement. But has he done so? Gentlemen, it is a lamentable fact that the profession of the State has been appealed to through the officers of every county society to take up this work, and it has not responded.

Now, if you believe in exercising the high privilege your profession gives you, let us have your co-operation individually and collectively. Society looks to us for a verification of the statements herein given. If you believe them true, you have an added responsibility to bear, and now, will you prove yourselves equal to it?

